# The Effects of Acute Tryptophan Depletion on Reactive Aggression in Adults with Attention-Deficit/Hyperactivity Disorder (ADHD) and Healthy Controls

**DOI:** 10.1371/journal.pone.0032023

**Published:** 2012-03-14

**Authors:** Marco Zimmermann, Marco Grabemann, Christian Mette, Mona Abdel-Hamid, Jennifer Ueckermann, Markus Kraemer, Jens Wiltfang, Bernhard Kis, Florian Daniel Zepf

**Affiliations:** 1 Department of Psychiatry and Psychotherapy, LVR-Clinics Essen, University of Duisburg-Essen, Essen, Germany; 2 Department of Neurology and Neurophysiology, Alfried-Krupp Hospital, Essen, Germany; 3 Department of Child and Adolescent Psychiatry, Psychosomatics and Psychotherapy, RWTH Aachen University, Aachen, Germany; 4 Institute for Neuroscience and Medicine, Jülich Research Centre, Jülich, Germany; 5 Jülich-Aachen Research Alliance (JARA) Translational Brain Medicine, Aachen and Jülich, Germany; University of New South Wales, Australia

## Abstract

**Background:**

The neurotransmitter serotonin (5-HT) has been linked to the underlying neurobiology of aggressive behavior, particularly with evidence from studies in animals and humans. However, the underlying neurobiology of aggression remains unclear in the context of attention-deficit/hyperactivity disorder (ADHD), a disorder known to be associated with aggression and impulsivity. We investigated the effects of acute tryptophan depletion (ATD), and the resulting diminished central nervous serotonergic neurotransmission, on reactive aggression in healthy controls and adults with ADHD.

**Methodology/Principal Findings:**

Twenty male patients with ADHD and twenty healthy male controls were subjected to ATD with an amino acid (AA) beverage that lacked tryptophan (TRP, the physiological precursor of 5-HT) and a TRP-balanced AA beverage (BAL) in a double-blind, within-subject crossover-study over two study days. We assessed reactive aggression 3.25 hours after ATD/BAL intake using a point-subtraction aggression game (PSAG) in which participants played for points against a fictitious opponent. Point subtraction was taken as a measure for reactive aggression. Lowered rates of reactive aggression were found in the ADHD group under ATD after low provocation (LP), with controls showing the opposite effect. In patients with ADHD, trait-impulsivity was negatively correlated with the ATD effect on reactive aggression after LP. Statistical power was limited due to large standard deviations observed in the data on point subtraction, which may limit the use of this particular paradigm in adults with ADHD.

**Conclusions/Significance:**

Together with previous findings, the data provide preliminary evidence of an inverse association between trait-impulsivity and the ATD effect on reactive aggression after LP (as assessed by the PSAG) in patients with ADHD and that this relationship can be found in both adolescents and adults. Because of limited statistical power larger sample sizes are needed to find main effects of ATD/BAL administration on reactive aggression in adults with ADHD.

## Introduction

Recent research has shown that symptoms related to attention-deficit hyperactivity disorder (ADHD), a disorder predominantly linked to changes in catecholaminergic neurotransmission, are also affected by variation in serotonin-related genes [1], [2], [3], [4]. There is a considerable body of evidence showing that neurobiological and environmental factors significantly contribute to disorders and phenotypes associated with aggression and impulsivity [2], [4]. The neurotransmitter serotonin (5-HT) has been linked to the neurobiological underpinnings of aggressive behavior by a considerable body of animal research, suggesting that low central nervous system 5-HT activity is associated with aggression and impulsivity [5], [6], [7].

A considerable amount of studies investigated the effects of changes in serotonergic neurotransmission on aggression in rodents. Evidence suggests that reduced serotonin-mediated inhibition facilitates aggressive behavior [8]. Moreover, prolonged TRP deprivation lead to increased killing of mice in rats when compared to standard foods that were administered to the animals [9]. However, the data of this particular study also suggested that mouse-killing persisted after rats were returned to standard food, indicating that reduced serotonergic neurotransmission did not influence the maintenance of aggression [9]. In addition, research using the selective and irreversible TPH2 inhibitor p-chlorophenylalanine (PCPA) indicated that depletion of central nervous serotonin function in rats increased nonspecific killing reactions but was insufficient as regards species-specific predatory behavior [10]. Forebrain structures and depletion of serotonin function by the neurotoxin 5,7-dihydroxytryptamine (5,7-DHT) in these brain areas were shown to be of particular relevance [11]. Finally, a further study using PCPA administration in rats showed that only minimal impairments as regards serotonin modulated inhibitory control resulted in muricidal aggression in rats of both sexes that was related to a depletion in central nervous serotonin function of approximately 55–60% [12]. Overall, the relationship between reduced central nervous serotonin synthesis achieved by different depletion techniques in rats and increased aggression is a rather robust finding [8], [9], [10], [11], [12], [13], [14], [15], [16]. Early acute tryptophan depletion (ATD) studies that were conducted in rats indicated significant effects on a diminished 5-HT synthesis [13], [17], [18].

A number of behavioral studies in humans have used the ATD technique to investigate impulsive and aggressive behavior after the central nervous 5-HT synthesis rate has been experimentally depleted [19], [20], [21], [22], [23]. Studies have used modifications of the “Point Subtraction Aggression Paradigm” (PSAP) [24] to assess reactive aggressive behavior under depleted and sham-depleted conditions. Several studies have detected increased aggression during diminished central nervous synthesis of 5-HT in healthy (mostly male) subjects [21], [22], [25]. However, ATD has also been shown to decrease aggression in nonaggressive men [21]. Salomon and colleagues found that that ATD did not affect aggression as assessed by the Buss-Durkee Hostility-Guilt Inventory and the Overt Aggression Scale (OAS) [26]. Moreover, LeMarquand and coworkers also did not detect a relationship between acutely diminished central nervous 5-HT synthesis and aspects of impulsive [27], [28] or impulsive and aggressive behavior [28] in either young adults with a family history of alcoholism [28] or in young, aggressive males [27] using a modified Taylor task [28] and a go/no-go learning task [27], [28]. Of note, the negative findings of LeMarquand et al. in their study of young, aggressive males was possibly due to a ceiling effect [27]; a subsequent study found a group-specific effect. Using a behavioral measure, only high-aggressive subjects showed increased aggression after ATD; low aggressive subjects showed no difference. Overall, both group-specific [20], [21], [22], [26], [29], [30] and task-related characteristics [27], [28] must be taken into account to explain the currently available results (for an overview, see [31]).

There is a scarcity of data on the relationship between 5-HT function and aggression in children and adolescents. Studying the developmental trajectory of reactive aggression in relation to clinical phenotypes is highly relevant to understanding the development of neuropsychiatric disorders associated with high levels of aggression and violence [32], [33]. Behavioral findings have shown that low-impulsive children and adolescents with ADHD behave in a highly impulsive and aggressive manner when subjected to ATD [34]. However, there are no studies to date that have investigated the effects of ATD on reactive aggression in adult patients with ADHD, a disorder that frequently presents with co-morbid aggression and behavioral problems.

In the present study, we investigated the effects of ATD compared with a tryptophan-(TRP)-balanced control condition (TRP balanced amino acid load, BAL) on reactive aggression, which we assessed with the Point-Subtraction Aggression Game (PSAG, using high provocation [HP] and low provocation [LP] trials) in a sample of male patients with ADHD and healthy controls.

## Results

### Between-group comparison

**Figure 1:**
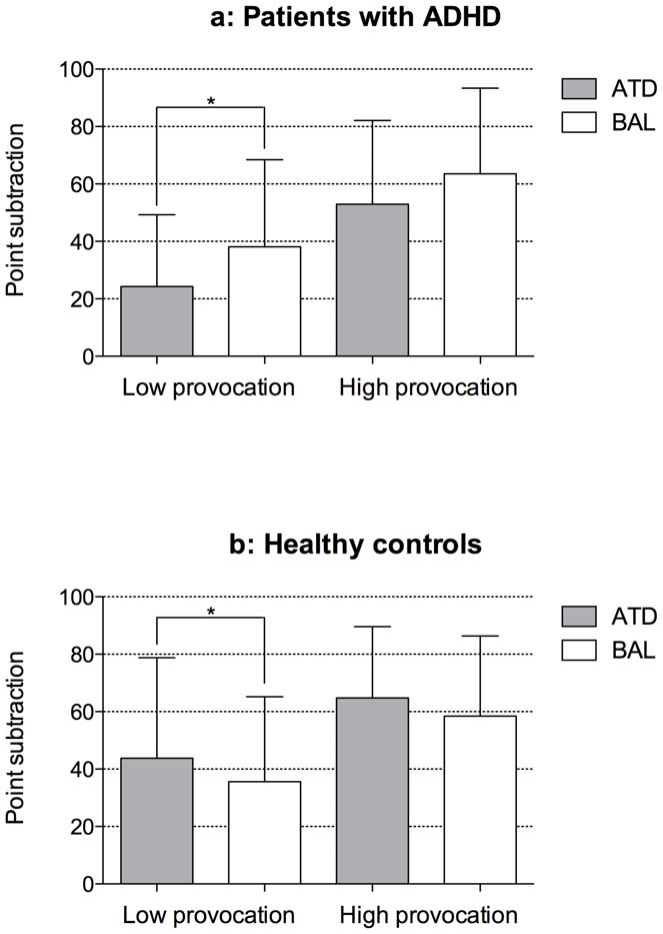
**Fig. 1a** **.** Mean point subtraction with standard deviation after low (LP) and high provocation (HP) in a point-subtraction aggression game. Data were obtained during a period of a diminished central nervous serotonin synthesis rate (Acute tryptophan depletion, ATD) and a control condition (Tryptophan balanced amino acid load, BAL). Significant relationships are indicated by * (*p*<0.05). **Fig. 1b** **.** Mean point subtraction with standard deviation after low (LP) and high provocation (HP) in a point-subtraction aggression game. Data were obtained during a period of a diminished central nervous serotonin synthesis rate (Acute tryptophan depletion, ATD) and a control condition (Tryptophan balanced amino acid load, BAL). Significant relationships are indicated by * (*p*<0.05).

A 2 (group: ADHD vs. controls)×2 (treatment: ATD vs. BAL)×2 (provocation: HP vs. LP) repeated measures analysis of variance (RMANOVA) showed a significant group-by-treatment interaction (*p* = 0.017, F = 6.256, df = 1, 39). Post-hoc analyses indicated that in patients, fewer points were subtracted in the LP condition during ATD than during BAL (t = 2.341, *p* = 0.030, df = 1, 19; see Fig. 1a). For the HP condition, we did not detect such a difference (t = 1.241, *p* = n.s., df = 1, 19). In the group of healthy controls, more points were subtracted in the LP condition during ATD than during BAL (t = 2.382, *p* = 0.028, df = 1, 19, see Fig. 1b). For the HP condition, we did not find such a difference (t = 1.318, *p* = n.s., df = 1, 19). Participants in both groups subtracted more points in the HP condition than in the LP condition (*p* = 0.000, F = 49.551, df = 1, 39). Subjects with a combined (n = 13) or inattentive (n = 7) subtype of ADHD did not differ in the ATD effect on point subtraction as a function of provocation (LP/HP) or treatment (ATD/BAL) (RMANOVA with no significant treatment [ATD vs. BAL]× sub-group interaction *p* = n.s., F = 0.940, df = 1, 39).

### Dimensional analysis

In the patient group, there was a significant negative correlation between the ATD effect on point subtraction (ATD minus BAL, hereafter Δ) in the PSAG after low provocation (ΔLP) and baseline impulsivity scores as indexed by the Barratt Impulsiveness Scale (BIS) Motor Impulsiveness subscale (MI; *Rho* = −0.506; *p* = 0.023), and MI explained approximately 25.6% of the shared intraindividual variance. ΔLP was also negatively correlated with the total impulsivity scale (TI; *Rho* = −0.448; *p* = 0.048), and explained approximately 20.1% of the shared intraindividual variance. The correlation between ΔLP and unplanned impulsivity (UI; *Rho* = −0.379; *p* = n.s.) and cognitive impulsiveness (CI; *Rho* = −0.148; *p* = n.s.) was not significant. In the control group, we found no such correlations with ΔLP (MI: *Rho* = −0.188, *p* = n.s., UI: *Rho* = −0.184, *p* = n.s.; CI: *Rho* = −0.275, *p* = n.s.; TI: *Rho* = −0.300; *p* = n.s.) or ΔHP (MI: *Rho* = −0.212, *p* = n.s., UI: *Rho* = −0.155, *p* = n.s.; CI: *Rho* = −0.132, *p* = n.s.; TI: *Rho* = −0.145; *p* = n.s.).

## Discussion

To our knowledge, this is the first study to investigate the effects of ATD on reactive aggression in adult patients with ADHD compared with healthy adult controls. Both groups subtracted more points after HP than LP regardless of ATD/BAL intake, which is consistent with the concept of PSAG, the behavioral paradigm used to assess aggressive behavior. Patients with ADHD subtracted fewer points under ATD than BAL in the LP condition, but the HP condition showed no difference. This effect was not found in our previous study of children and adolescents with ADHD [34], suggesting a potential developmental difference in the total number of points subtracted under depletion conditions. However, in the subgroup of patients with ADHD, there was a negative correlation between trait-impulsivity scores (in particular motor impulsivity) and ΔLP, which is consistent with the data we have previously obtained in children and adolescents with ADHD [34]. This finding is of particular relevance as it suggests that both adolescent and adult low trait-impulsive patients with ADHD may respond with increased reactive aggression under ATD after a low provocation [32]. In addition, low-impulsive patients in particular showed a lowered heart rate under ATD [35] while behaving aggressively [34]. This is consistent with previous findings showing that the low provocation condition in the used behavioral paradigm is the most effective to assess anger reactive responses [36], [37]. A negative correlation was found between baseline aggression scores and the ATD effect on extraversion ratings of a fictitious opponent in adolescents with ADHD, which supports the idea that there is an inverse association between changes in 5-HT neurotransmission and impulsivity/aggression-related behavioral traits [38]. From this, one could conclude that low-impulsivity patients with ADHD are particularly susceptible to an increase in reactive aggression when the central nervous 5-HT synthesis rate is diminished.

The present study has several significant advantages over previous research. First and foremost, apart from the double-blind within-subject repeated measures design it included a healthy control group that did not differ from the patients in education, IQ or age, which was not possible in previous research [34], [39], [40], [41]. Second, using the same behavioral paradigm as previous research with children and adolescents allows us to look at the behavioral data from a developmental viewpoint as part of an iterative approach that focuses on the neurobiological underpinnings of anger reactive aggression in ADHD. Moreover, the same neurochemical depletion procedure (ATD Moja-De) was used as in studies on children and adolescents [34]. In addition, the used depletion paradigm ATD Moja-De takes the body weight of the subjects as well as their baseline TRP into account (as there are correlations between body weight and baseline tryptophan, see [42]), resulting in improved tolerability and allowing its use in children and adolescents. Finally, the administration of ATD/BAL was designed as a within-subject repeated measures factor in a double-blind setting, allowing sufficient statistical power to detect significant differences in mean point subtraction in the PSAG under ATD between the two groups. Cohen's d values as an estimate for effect sizes were moderate as regards the difference in mean point subtraction under ATD between the two groups.

There are some methodological considerations to be aware of when trying to interpret data related to reactive aggression obtained using the PSAG under ATD/BAL. First and foremost, the PSAG task as used in the present study did not allow the participants to not behave aggressively [31], [34]. In particular, as regards HP trials in terms of a ceiling effect subjects might have felt to have to subtract as many points as their opponent did, whereas for the LP condition there was a greater range to over-respond or overreact to the received provocation. This is in line with past research using the PSAG and its modifications, indicating that the LP condition is particularly sensitive to assess an ager reactive response [36], [37]. Second, using the adapted version for adolescents in adults might have caused altered response patterns (as indicated by the rather large standard deviations in the PSAG data in the adult subjects). However, this finding is of value for future research aiming to use behavioral paradigms to assess aggressive behavior in both youths and adults from a developmental viewpoint. As elucidated by the present study further research on the validity of using this particular version of the PSAG in adults is warranted. Third, a drawback to the study is that there are no data from females, which questions whether the results would be found in female patients with ADHD. This concern is relevant because past research has indicated that there are significant differences in 5-HT brain synthesis between males and females [43]. Moreover, genetic data regarding the serotonin system were not obtained, which would have resulted in significant larger sample sizes. Further research with significantly larger samples is warranted to detect whether variation in genes linked to serotonergic neurotransmission impact reactive aggression assessed under a diminished central nervous 5-HT synthesis rate.

In summary, the present data provide supporting preliminary evidence for an inverse association between measures of trait-impulsivity and the ATD effect on reactive aggression in patients with ADHD, and this relationship can be found in adolescents and adults. However, sample size was too small in order to be able to find significant main effects of the factor ATD/BAL on reactive aggression in adult patients with ADHD. More research on the laboratory assessment of reactive aggression in adults using the PSAG and studies involving females are needed.

## Materials and Methods

### Sample

We recruited twenty adult male patients with a confirmed diagnosis of ADHD (using DSM-IV criteria) from the outpatient clinic of the Department of Psychiatry and Psychotherapy at the University of Duisburg-Essen. The diagnosis of ADHD in each patient was also confirmed using the Wender Utah Rating Scale (WURS, [44]). Thirteen patients were diagnosed with the combined ADHD subtype and seven patients were diagnosed with the inattentive subtype. Other psychiatric disorders, such as co-morbid depression and mood disorders or substance abuse, as well as the current use of psychotropic medications other than methylphenidate (MPH) were exclusion criteria. They were ruled out by interview and anxiety symptoms were ruled out using the German Version of the State-Trait Anxiety Inventory [45]. Twenty healthy adult male controls were recruited from various university and non-university settings. No participant had a history of obesity, asthma, diabetes, substance abuse, psychotic disorders, or neurological diseases. During testing, patients were free from all medication. MPH (n = 16 patients) was not taken for one day before each study day, or two days prior to the study day in the case of patients prescribed long-acting MPH-based formulations. On both study days, participants were asked before they participated to confirm they had not taken their MPH medication. Participants also had to confirm that they did not take any other recreational drugs. Between the two study days, MPH was taken as prescribed. As regards medication history, one patient took atomoxetine before the study but medication was stopped several weeks before participation in the trial. One patient took an antidepressant (SSRI) before the trial and treatment was stopped several weeks before the study. Two patients had received a combination of MPH and antidepressants (SSRI/SNRI), and antidepressive treatment was stopped several weeks before participation. All patients were instructed not to consume caffeine or nicotine on study days, and regular heavy smokers (>8 cigarettes per day) were excluded from the study. We assessed cognitive abilities with the Wechsler Adult Intelligence Scale (3^rd^ edition), and found no significant group differences (*p* = n.s., t = −6.098, F = 4.972, df = 1, 38; see Table 1). No subject was accepted with an IQ below 75. A complete study description was handed out to all participants, and informed written and verbal consent were obtained. The study was assessed and approved by the Ethics Commission of the Faculty of Medicine of the University Duisburg-Essen, and was carried out in accordance with the Declaration of Helsinki.

**Table 1:** The characteristics of the study sample (a group of patients with attention-deficit/hyperactivity disorder (ADHD) and a group of healthy controls).

	Patients with ADHD (n = 20)			Healthy controls (n = 20)			
	M ± SD	Min.	Max.	M ± SD	Min.	Max.	*p*
**Age**	30.25±9.37	19	46	27.90±6.02	20	46	*n.s.*
**IQ**	108±15.875	77	130	109±10.65	96	136	*n.s.*
**Education (yrs)**	13.90±3.40	8	20	15.25±2.83	10	21	*n.s.*

Age on the first day of the study, IQ (as assessed by the Wechsler Adult Intelligence Scale [3rd edition]), and years of formal education. Mean values were compared, using two-tailed t-tests, to identify significant differences between adult patients with ADHD and healthy adult controls.

### Study design

The study used a double-blind within-subject crossover design. ATD/BAL amino acid (AA) administration was randomized and counterbalanced. In accordance with Moja and colleagues [46], subjects received an AA beverage lacking TRP (the ATD condition), which was modified for the impact of body weight [31], [34], [47], on one day. On another day, they received a TRP-balanced amino acid load (BAL) as a control condition (sham depletion). Testing days were spaced seven days apart. In both groups, ten participants received the ATD beverage on the first day of the study, and ten participants received it on the second. Both the patients and the investigator were blind to the order of ATD/BAL administration. On each testing day, 3.25 hours after administration of ATD/BAL (time point of approx. 90% reduction in 5-HT synthesis rate, see [42]), patients were administered the Point Subtraction Aggression Game (PSAG), a task designed to provoke reactive aggressive behavior. The PSAG has been employed in previous research on children and adolescents with ADHD to assess anger reactive aggression [34], [37]. Subjects were debriefed after completing the study.

### Depletion procedure

Subjects were required to maintain a protein-free diet after 8 p.m. on the day before each testing session. The AA mixtures for the two conditions looked optically identical. We used a fixed standardized version for a normal body weight of 80 kg in accordance with the Moja-De ATD protocol [48]. The AAs were mixed with 150 ml of water prior to administration. Immediately after intake of ATD/BAL, subjects were given a multivitamin candy to counteract the AA mixture's sour taste. One hour after ATD/BAL administration, all participants received a multi-vitamin beverage containing 30% of the recommended daily dose of nicotine adenine dinucleotide, which is a TRP-derived vitamin [47].

### Assessment of trait-impulsivity

Motor Impulsiveness (MI), Cognitive Impulsiveness (CI) and Nonplanning Impulsiveness (NI), as well as total impulsivity (TI), were assessed using the Barratt Impulsiveness Scale (BIS) on the first day of the study [49].

### Assessment of reactive aggression

The Point Subtraction Aggression Game (PSAG) is a modified version of the PSAP [24] that was developed for use in patients with disruptive behavior disorders [37]. In this task, subjects compete for points with a fictitious opponent of the same gender. We told subjects that they would be playing against a clinic staff member who was sitting in a different room of the building and that his computer was linked to the subject's computer via a local area network (LAN). The investigator called the staff member by phone to clarify relevant details for playing the game via LAN, such as the subject's identity number, etc.

The task was to react to a visually presented stimulus faster than the opponent by pressing a computer button. The stimulus was a soccer ball, and it appeared in a randomized order from different locations of the computer screen. To increase the difficulty, the time interval between the start of a new trial and the appearance of the stimulus was also randomized. The subject was instructed to achieve a high score at the end of the game. He was awarded 50 points on winning trials and had 50 points subtracted on losing trials. Additionally, the opponent had the opportunity to subtract between 0 and 100 points in steps of 10 points from the subject if he lost a trial. Conversely, if the subject won a trial, he could deduct between 0 and 100 points from his opponent. Winning and losing trials were predetermined within the game, and the sequence of winning and losing trials was the same on both testing days.

The dependent variable was the number of points that the subject deducted from his opponent after being provoked with a subtraction of a certain number of points by the opponent. The level of provocation was defined by the number of points the opponent subtracted from the subject. A high-provocation (HP) level was defined as an 80 to 100 point subtraction, and a low-provocation (LP) level was defined as a 20 to 40 point subtraction. The subject's reactive aggression was assessed using the number of points he subtracted from the opponent on a winning trial following a losing trial, with a certain level of provocation (HP/LP). The game lasted 48 trials with a 1-minute break after 24 trials. Before the game started, the participants were asked if they were nauseous and if they agreed to complete a short in-house questionnaire on how they felt before and after the PSAG.

### Data analysis

The normality of the data was analyzed using Kolmogorov–Smirnov's ‘Goodness of fit test.’ The point subtraction data in the PSAG were normally distributed. Because of an explorative approach significant *p*-values were not subjected to alpha-adjustment. The level of statistic significance was set at *p*<0.05. ATD/BAL intake effects on reactive aggression as a function of provocation level and group were analyzed using a repeated measures analysis of variance (RMANOVA) with group (ADHD/controls), treatment (ATD/BAL), and provocation (HP/LP) as factors. Additional post-hoc analyses (t-tests for additional within-group comparisons of point subtraction under ATD and BAL) and between-group comparisons of descriptive sample characteristics data (Table 1) were performed. The relationship between trait-impulsive characteristics, as assessed by the BIS, and reactive aggression under ATD was examined using Spearman rank correlations (*Rho*) between MI, CI, UI and the difference in point subtraction from ATD to BAL intake (Δ = Delta) for both provocation levels (ΔLP, ΔHP). Spearman's rank correlations (*Rho*) were used because they are very robust to outliers, as the analysis of data is independent of its distribution and the correlation coefficient is invariant to transformation. Potential effects of diagnostic characteristics (age, IQ, diagnosis, and BIS total impulsivity) on point subtraction (HP/LP) were controlled for using a two-way analysis of covariance with repeated measures (RMANCOVA) with the characteristics as covariates. The RMANCOVA was non-significant for all covariates. We also tested for potential order effects (ATD on day 1 vs. ATD on day 2) on point subtraction (HP/LP) in the whole sample. A two-way RMANOVA (with ATD on day 1 vs. day 2 as a between-subject factor) performed for this purpose showed no significance (the interaction effect for ATD administration and HP/LP; F = 0.059, *p* = n.s., df = 1, 39). Moreover, there were no significant differences between the different groups of patients who received different medications for treatment of ADHD symptoms before the study on point subtraction as a function of treatment (ATD/BAL) or provocation (LP/HP) as indexed by a RMANOVA with no significant medication group × treatment (ATD/BAL) interaction (*p* = n.s., F = 0.113, df = 1, 39). We calculated Cohen's d values for each level of provocation (HP/LP) as an estimate for effect sizes with respect to detecting a difference in mean point subtraction under ATD between groups, indicating a moderate effect sizes for both provocation levels (d_HP_ = 0.438; d_LP_ = 0.646). Based on the calculated effect sizes we also conducted a post-hoc power analysis using G Power Software (University of Kiel) in order to determine the statistical power of the completed experiment. We detected a power (1-β err prob) of 0.99 for the LP condition and a power of 0.97 for HP trials to detect significant between-subject differences in means with an alpha level of 0.05 in a RMANOVA. Following this, the present study was adequately powered.
