# Correction: The Effects of Acute Tryptophan Depletion on Reactive Aggression in Adults with Attention-Deficit/Hyperactivity Disorder (ADHD) and Healthy Controls

**DOI:** 10.1371/annotation/e575c960-6acb-47fc-af2b-212f13164aaf

**Published:** 2012-03-23

**Authors:** Marco Zimmermann, Marco Grabemann, Christian Mette, Mona Abdel-Hamid, Jennifer Uekermann, Markus Kraemer, Jens Wiltfang, Bernhard Kis, Florian Daniel Zepf

There was a spelling error in the fifth author's name. The correct spelling is Jennifer Uekermann. The correct citation is: Zimmermann M, Grabemann M, Mette C, Abdel-Hamid M, Uekermann J, et al. (2012) The Effects of Acute Tryptophan Depletion on Reactive Aggression in Adults with Attention-Deficit/Hyperactivity Disorder (ADHD) and Healthy Controls. PLoS ONE 7(3): e32023. doi:10.1371/journal.pone.0032023 

